# Activating empowerment through AI-guided reflection: the role of authentic leadership cues in a randomized controlled experiment

**DOI:** 10.1186/s40359-026-04537-y

**Published:** 2026-04-14

**Authors:** Indrayanti Indrayanti, Faturochman Faturochman, Muhammad Idham Ananta Timur, Karin Sanders, Fatimah Fatimah, Muaiyadah Muaiyadah

**Affiliations:** 1 Faculty of Psychology, Gadjah Mada University, Sosio Humaniora 1 Bulaksumur, Yogyakarta, 55281 Indonesia; 2 Department of Computer Sciences and Electronics, Faculty of Mathematics and Natural Sciences, Gadjah Mada University, Sekip Utara BLS 21, Yogyakarta, 55281 Indonesia; 3 School of Management and Government, Business School, University of New South Wales, Level 5, West Wing, Sydney, Australia

**Keywords:** Psychological empowerment, Leadership priming, Reflective chatbots, AI-mediated reflection, Digital workplace, Employee well-being, Narrative analysis, Collectivist culture

## Abstract

**Background:**

Effective employee empowerment in digital workplaces necessitates reflective interventions that are psychologically grounded and scalable. Leadership related cues can activate cognitive schemas, thereby influencing individuals’ interpretations of subsequent experiences. However, limited research has explored the interaction between such cues and AI-guided reflection. This study investigates the effects of exposure to authenticity-related leadership cues, employed as a priming stimulus, in conjunction with chatbot-guided reflection chatbots on psychological empowerment narratives and mental well-being.

**Methods:**

A randomized controlled trial was conducted with 53 Indonesian employees, who were assigned to either a high-authenticity priming condition (EG-1), a low-authenticity priming condition (EG-2), or chatbot-only control condition (CC). After video-based priming, all participants engaged in structured chatbot reflections across four empowerment dimensions: meaning, competence, self-determination, and impact. Quantitative well-being outcomes and qualitative narrative patterns were examined using a mixed method approach.

**Results:**

Participants in EG-1 showed significantly greater improvements in well-being (e.g., workplace well-being + 4.50, d = 1.89; general well-being + 3.88, d = 1.58), while EG-2 exhibited declines. Qualitative analysis revealed that reflections in the EG-1 were more self-attributed, emotionally elaborated, and meaning-oriented, in contrast to the more externally anchored or affectively neutral narratives observed in the EG-2 and CC.

**Conclusions:**

The findings indicate that authenticity-related leadership cues serve as psychological prime, shaping the interpretive context of AI-guided reflection. Reflective chatbot offers a psychologically safe medium for narrative sensemaking. This combination presents a context-sensitive strategy for fostering empowerment and well-being in collectivist and high power-distance workplace environments.

**Supplementary Information:**

The online version contains supplementary material available at 10.1186/s40359-026-04537-y.

## Introduction

Psychological empowerment has become increasingly important in today’s digital workplace, where employees must navigate complex, technology-driven environments. Defined by meaning, competence, self-determination, and impact [1], empowerment is linked to motivation, well-being, and adaptability. However, the shift toward hybrid and distributed work limits consistent face-to-face leadership interactions, raising the question of how empowerment can be cultivated through scalable psychological mechanisms.

Recent work links empowerment and related constructs to meaningful work and employee well-being in contemporary, digitally demanding contexts [2]. At the same time, narrative and reflective perspectives highlight empowerment as a discursive process constructed through meaning-making [3]. From this view, employees articulate and negotiate their sense of agency through the stories they tell about competence, autonomy, and impact.

Building on this narrative orientation, leadership becomes a crucial interpretive anchor. Authentic leadership, in particular, has attracted substantial attention for its emphasis on self-awareness, relational transparency, balanced processing, and an internalized moral perspective [4]. These qualities help employees interpret work as meaningful and reinforce competence and agency.

At the same time, critical scholarship has cautioned against overly idealized and normatively positive portrayals of authentic leadership. Scholars argue that this construct is vulnerable to conceptual ambiguity, moralization, and the oversight of power relations, contextual influences, and potential dysfunctional consequences [5, 6]. From this perspective, authentic leadership should be regarded not as an unproblematic or universally advantageous model, but as a contested and context-dependent leadership narrative.

In the present study, authentic leadership is not conceptualized as a prescriptive ideal or a stable leader trait. Rather, it is considered a symbolic and psychological cue that may activate specific interpretive schemas related to trust, transparency, and agency during reflective processes. In digital and hybrid workplaces, however, such cues may be brief, indirect, or inconsistent, limiting opportunities for sustained influence. Accordingly, the present study does not test the validity, desirability, or ethical superiority of authentic leadership as a leadership model but only examines the psychological effects of exposure to authenticity-related cues during reflection.

From a cognitive perspective, leadership cues activate internalized schemas that guide individuals’ interpretation of subsequent experiences [7]. Signals of authenticity, including relational warmth and transparent communication, influence immediate trust and motivation as well as the processing and reflection on later events. Experimental studies indicate that even brief exposure to leaders’ visual and relational cues can shape perceived suitability, affective trust, and relational engagement [8–10]. Collectively, this literature suggests that minimal leadership cues may influence how individuals approach subsequent reflective tasks. Notably, schema activation does not indicate that the leadership model itself is valid or superior; rather, such cues serve as psychologically meaningful interpretive frames.

Parallel to these leadership developments, digital tools are beginning to shape employee engagement and well-being. Interventions such as gamification, e-coaching, and AI-driven chatbots are increasingly used to support reflection and emotionally safe self-disclosure [11–13]. Reflective chatbots, in particular, offer a unique opportunity: as neutral and nonjudgmental partners, they can guide employees through structured introspection, eliciting narratives about work and personal agency that might otherwise remain unspoken. However, little is known about how such reflective technologies interact with leadership cues, especially those related to authenticity, to shape the way employees narrate their empowerment experiences.

Systematic reviews further show that AI-based conversational agents promote reflective thinking and emotional openness [14] and provide scalable psychological support [15]. These properties make chatbots particularly suitable for contexts where direct interpersonal disclosure may feel risky or culturally constrained.

The cultural context sharpens the relevance of this investigation. Indonesia, as a high power-distance and collectivist society, is characterized by hierarchical norms that may inhibit open self-expression, particularly around issues of autonomy, competence, and impact [16–18]. In such contexts, employees may hesitate to voice empowerment-related experiences directly to leaders for fear of disrupting harmony or appearing disrespectful. Digital reflection with chatbots may provide a culturally attuned medium to bypass these constraints, allowing employees to articulate empowerment narratives with greater safety and authenticity. By situating this study in Indonesia, we highlight how leadership–technology synergies can unfold differently in non-Western, collectivist work cultures, where empowerment may be experienced but not always openly expressed.

From a practical perspective, understanding the interaction between leadership priming and reflective chatbots has significant implications for organizations aiming to implement scalable strategies to enhance employee well-being and empowerment in digital work environments. Chatbot-mediated reflection, when integrated with leadership cues, may serve as a cost-effective and accessible supplement to traditional leadership development and employee support, especially in settings where coaching or mental health resources are limited.

To address these gaps, this study integrates authentic leadership priming with AI-guided reflective chatbot interactions. We adopt a mixed-methods design that examines not only quantitative changes in well-being but also the narratives employees construct about their empowerment. This approach positions empowerment as both a psychological state and a narrative process emerging during reflection.

Accordingly, the present study makes three contributions. First, it examines how authentic leadership priming shapes digital narrative empowerment. Second, it advances understanding of leadership–technology synergy in collectivist, high power-distance work cultures, offering insights that challenge Western-centric assumptions of empowerment. Third, it demonstrates a scalable and culturally responsive intervention model that supports empowerment and well-being in digital workplace contexts.

## Theoretical background & hypotheses

### Leadership priming and schema activation

Leadership is widely recognized as a social influence process that shapes individuals’ thoughts, emotions, and behaviours within organizational contexts. In addition to behavioural interactions, social cognitive perspectives highlight that leadership operates symbolically through visual, verbal, and relational cues that activate internalized schemes and interpretive frames [10, 19]. These cues such as tone of voice, emotional expression, and relational posture, are often processed automatically and can influence perceptions of authority, trustworthiness, and personal role within organizations.

Experimental priming research demonstrated that even brief exposure to leadership-related stimuli can activate mental representations of leadership archetypes, subsequently shaping cognitive and affective processes such as trust, motivation, and self-reflection. In the leadership literature, authentic leadership, is typically conceptualized through the dimensions of self-awareness, relational transparency, balanced processing, and an internalized moral perspective [4], has become a prominent discourse in the leadership literature and is frequently used in empirical studies examining empowerment, trust, and well-being [20].

Concurrently, this research stream has generated substantial conceptual and critical debate. Scholars have questioned the coherence, moralization, and normative assumptions embedded to oversimply power relations, contextual factors, and the potential dysfunctional consequences of “being authentic” [5, 6]. These critiques indicate that authentic leadership should not be regarded as an unproblematic or universally desirable leadership model.

Consistent with this critical perspective, the present study does not conceptualize authentic leadership as a prescriptive ideal, a stable leader trait, or a theory requiring validation. Instead, authenticity-related cues are regarded solely as symbolic and psychological stimuli for priming. From a schema-based perspective, these cues may activate interpretive frames related to trust, openness, and agency, thereby influencing individuals’ approaches to subsequent reflective tasks. This approach does not imply that authentic leadership constitutes a coherent, sufficient, or normatively superior theory of leadership, but rather acknowledges that authenticity-related signals serve as psychologically meaningful inputs within sensemaking processes.

Empirical research indicates that exposure to relationally supportive or transparent leadership cues can influence affective trust and openness in leader-follower relationships [21, 22]. However, considerably less is known about how these cues shape reflective processing, particularly when reflection is mediated by digital tools designed to scaffold narrative construction.

This study addresses this gap by examining how exposure to authenticity-related leadership cues, used exclusively as a priming device, shape the articulation of psychological empowerment during chatbot-mediated reflection. Drawing from [1] model, the analysis focuses on meaning, competence, self-determination, and impact, and proposes that leadership-related primes influence not the existence of empowerment, but rather how individuals frame, interpret, and narrate their experiences during reflection.

### Reflective chatbots and AI-mediated reflection

Conversational agents are increasingly utilized in mental health, education, and organizational settings to support self-reflection and structured self-disclosure. According to the Computers Are Social Actors (CASA) paradigm, users often respond to chatbots as if they were social partners engaging in disclosure, empathy, and emotional engagement [23, 24]. Research shows that reflective chatbots can enhance awareness, promote emotional insight, and support well-being when they use empathic prompts or reciprocal disclosure [12, 25, 26]. In line with this, high perceived social presence reliably predicts greater depth and willingness of self-disclosure to conversational AI [27].

Unlike informational bots, reflective chatbots function as scaffolds for narrative elicitation, enabling individuals to articulate meaning, agency, and competence in ways that may remain latent in face-to-face contexts. In this study, chatbot prompts were designed in [1] four dimensions of empowerment, shifting from static survey responses toward narratively constructed reflections.

Crucially, the effectiveness of chatbot-guided reflection depends not only on prompt design or interface features, but also on the user’s psychological readiness and interpretive framework. The same reflective scaffold can elicit either superficial or deeper engagement, contingent upon individual approaches to the task. Leadership priming may function as a preparatory mechanism that shapes the tone, depth, and ownership of chatbot-elicited narratives. This study proposes a dual-level process in which leadership-related schemas influence interpretive framing, while the chatbot provides a psychologically safe, structured medium for articulating reflections. Thus, the chatbot is conceptualized not as an intervention, but as a facilitator of sensemaking.

### Psychological empowerment as a narrative construct

Psychological empowerment (PE) is traditionally defined as an intrinsic motivational state encompassing meaning, competence, self-determination, and impact [1]. It is typically assessed through self-report scales that capture individuals’ subjective evaluations of these dimensions. Recent scholarship, however, contends that empowerment should be understood not only as a measurable state but also as a discursive and interpretive process embedded in how individuals narrate their work, agency, and influence [3, 28].

Within narrative psychology, empowerment is understood not as a fixed psychological attribute, but as a dynamic meaning-making process enacted through reflection and language [9, 29]. This approach redirects analytical attention from the question of whether employees “possess” empowerment to the ways in which they construct and position themselves in relation to their work, competence, and perceived impact. Consequently, narrative analysis provides a process-sensitive framework for investigating how empowerment is articulated and negotiated during reflective practice.

By integrating leadership priming with chatbot-mediated reflection, this study advances empowerment research beyond a predominantly attitudinal measurement tradition toward a more process-oriented and narrative-cantered understanding of agency. This approach complements, rather than replaces, quantitative assessment by elucidating how empowerment is articulated, negotiated, and framed during reflective sensemaking.

Importantly, in everyday work narratives, the meaning dimension of psychological empowerment is often conveyed through how individuals connect routine tasks to personal values, identity, or long-term goals. For example, an employee may characterize an administratively demanding job not merely as “busy work,” but as meaningful because it provides opportunities to support their family or achieve professional growth. Such narrative framings illustrate both the cognitive appraisal of task significance and the active construction of meaning through self-interpretation. In this sense, psychological empowerment functions not only as an internal state but is also continuously enacted and negotiated through narrative sensemaking.

### Culture, power distance, and empowerment expression

Although psychological empowerment is frequently conceptualized as a universally relevant construct, its manifestation is significantly influenced by cultural context. In collectivist and high power-distance societies such as Indonesia, individuals are socialized to prioritize harmony, respect hierarchical authority, and regulate self-expression in accordance with social and relational norms [16–18]. Cultural mindset theory posits that these norms function as cognitive frames, shaping perceptions of what is appropriate, feasible, or risky to express in specific social contexts [30].

Within these contexts, overt demonstrations of autonomy, influence, or personal impact are often constrained, as direct self-assertion may be regarded as inappropriate or disruptive in hierarchical work environments [31]. These cultural scripts establish critical boundary conditions for empowerment interventions, which are often developed on implicit Western assumptions about open self-expression and individual voice.

Reflective chatbots may partially address these constraints by providing a psychologically neutral and less socially evaluative context for reflection, rather than eliminating cultural norms. The relative anonymity and nonjudgmental stance of chatbots can reduce the perceived interpersonal risk associated with expressing personal thoughts, doubts, or aspirations. When integrated with leadership cues that symbolically legitimize agency, such digital reflection may facilitate employees’ exploration of empowerment-related narratives that might otherwise remain unexpressed.

Therefore, the integration of leadership priming and AI-mediated reflection should be conceptualized as both a socio-cultural and technological mechanism. Its potential effectiveness stems from engaging with rather than circumventing cultural norms to establish safer conditions for sensemaking and self-articulation within hierarchical organizational environments.

### Integration and hypotheses

The framework does not presuppose that authentic leadership is universally effective, normatively superior, or free from theoretical challenges. Instead, it investigates how exposure to authenticity-related cues may serve as psychological and narrative resources that influence individuals’ approaches to reflective tasks within defined experimental and cultural contexts.

Synthesizing these perspectives, it is proposed that leadership priming and chatbot-guided reflection jointly influence the conditions under which empowerment-related sensemaking occurs. Leadership cues are expected to activate schemas related to trust, transparency, and agency, thereby shaping psychological readiness and interpretive framing. The chatbot provides a structured and minimally evaluative scaffold that facilitates the articulation of experiences through narrative reflection. Notably, this framework conceptualizes leadership cues and chatbots not as direct interventions that generate empowerment or well-being, but rather as contextual and procedural factors that influence how reflection is undertaken and expressed.

From this perspective, the combination of leadership priming and AI-mediated reflection is understood as a dual-pathway sensemaking arrangement rather than a prescriptive intervention model. This approach is particularly relevant in collectivist and high power-distance contexts, where cultural norms may otherwise limit open self-expression and agency-related narratives.

Based on this framework, the following hypotheses are formulated:

- H1: Exposure to high authentic leadership priming will lead to stronger narrative expressions psychological empowerment (meaning, competence, self-determination, and impact) compared to the low authentic leadership and control conditions.
- H2: Participants in the high authentic leadership condition will demonstrate greater improvements in post-intervention well-being (purpose in life, workplace well-being, general well-being, and reduced psychosomatic complaints) compared to the other groups.
- H3: Chatbot-guided reflection will elicit empowerment-related narratives across all conditions; however, reflections in the high-authentic leadership condition are expected to exhibit greater depth, elaboration, and self-attribution, indicating relatively stronger narrative empowerment patterns

To clarify the conceptual logic of this study, Table 1 summarizes how the theoretical lenses inform the proposed hypotheses. Leadership priming, grounded in social cognitive theory, is expected to shape interpretive readiness by activating schemas. Reflective chatbots, drawing on the CASA paradigm, provide a structured and psychologically low-risk medium for narrative articulation. Psychological empowerment is conceptualized as both a cognitive and narrative construct, while cultural norms of collectivism and power distance shape how such narratives are expressed and constrained. Together, these elements define the hypothesized dual pathway through which leadership cues and AI-mediated reflection jointly structure reflective sensemaking.

**Table 1:** Conceptual framework linking leadership priming, chatbot reflection, and empowerment outcomes

Theoretical Lens	Key Mechanism	Expected Narrative/Behavioural Expression	Hypothesis
Leadership Priming (Social Cognitive Theory) [19]	Authentic leadership cues activate schemas of trust, transparency, and agency.	Heightened readiness for self-reflection; positive appraisal of role and values.	H1
Reflective Chatbots (CASA Paradigm) [32]	Chatbots act as social partners, providing safe scaffolding for self-disclosure.	More elaborated and emotionally engaged narratives across empowerment dimensions.	H3
Psychological Empowerment [1]	Empowerment articulated through meaning, competence, self-determination, and impact.	Narratives show deeper ownership, agency, and future orientation.	H1, H3
Cultural Context (Collectivism & High-Power Distance) [16]	Hierarchical norms constrain direct expression; safe digital reflection reduces risk.	Empowerment expressed through culturally adapted narratives, enabled by chatbot neutrality.	H2, H3
Integration	Leadership priming + chatbot reflection as dual pathway.	Leadership cues shape framing; chatbot scaffolds articulation.	H1–H3

## Methods

### Design

A convergent mixed-methods experimental design was employed, integrating a randomized controlled trial (RCT) with qualitative narrative analysis. This approach enabled the capture of both outcome-level changes in well-being and process-level dynamics of psychological empowerment as reflected in participants’ narratives. Quantitative and qualitative data were collected during the same experimental session and analysed in parallel, facilitating complementary and mutually informative interpretations.

The quantitative component assessed changes in well-being indicators across experimental conditions, providing evidence of differential intervention effects. Concurrently, the qualitative component examined how participants articulated meaning, agency, competence, and impact in their chatbot reflections, offering insight into the psychological processes underlying these changes. Integration occurred at the interpretation stage, where patterns in narrative structure, depth, and attribution were analysed alongside group differences in well-being outcomes.

Participants were randomly assigned to one of three conditions: (1) High Authentic Leadership priming (EG-1) (2), Low Authentic Leadership priming (EG-2), or (3) a chatbot-only control condition (CC). The study was conducted online between September and October 2024 to enhance ecological validity in digital workplace settings. Each participant completed the study individually via a secure online platform, minimizing social desirability and peer influence during reflection.

### Participants

A total of 67 Indonesian employees (aged 25–50 years, M = 37.2, SD = 6.5; 53% female) were recruited via professional networks and online outreach. Eligibility criteria included at least 6 months of full-time employment. Baseline well-being was measured but did not serve as an inclusion or exclusion criterion. Following data cleaning, the final analytic sample comprised 53 participants (EG-1 = 17, EG-2 = 18, CC = 18).

Of the 67 employees recruited, 14 were excluded from the final analysis for not meeting eligibility criteria, such as having less than 6 months of full-time employment or submitting incomplete data. The sample size was determined based on organizational feasibility for a pilot experimental study, targeting approximately 50 active employees. This sample size was considered a pragmatic balance between feasibility and the ability to explore preliminary patterns in work-related experiences, given the intensive nature of the reflection-based procedure.

Randomization produced groups that were broadly comparable in age, gender, and baseline well-being indicators. All procedures received approval from an institutional ethics committee, and informed consent was obtained digitally. Monitored risks included emotional discomfort during reflection, fatigue, and concerns regarding data privacy in relation to management. Participants received a small appreciation package, including transport reimbursement, a souvenir, and light refreshments. These items were intended to support participation and were not performance-based incentives.

### Procedure

Recruitment was conducted through the organization’s official channels, but responses were submitted privately to the research team, ensuring that management could not identify who chose to participate or decline. Participation was entirely voluntary and had no consequences for employment status. Participants were then assigned to the three study conditions using a computer-generated random number sequence provided by the assistant researcher.

The study comprised five main stages:

1. Chatbot Needs Assessment: The chatbot design was informed by preliminary Focus Group Discussions (FGDs) with employees to identify user needs and interaction patterns.
2. Randomization Procedure: Participants were then assigned to the three study conditions using a computer-generated random number sequence provided by the lead researcher.
3. Pre-test survey: Participants completed validated measures of psychological empowerment and well-being.
4. Leadership priming: Depending on condition, participants viewed a 3-min video depicting high or low authentic leadership or a neutral workplace scene. Videos were scripted in Indonesian, acted by trained professionals, and validated by nine subject-matter experts using Aiken’s V (High = 0.856; Low = 0.789). A neutral workplace video was used for the control group.
5. Chatbot reflection. Immediately following the priming procedure, participants engaged in an approximately 30-minute session with a culturally adapted AI chatbot. To promote methodological transparency and replicability, the system-level prompt, interaction constraints, and the prompt framework are detailed in Appendix A. The chatbot adhered to a fixed interaction protocol: it began with a standardized opening question, posed only one question per turn, maintained a friendly and non-evaluative tone, and followed a predefined thematic progression aligned with Spreitzer’s four empowerment dimensions. Although the specific wording of follow-up questions was dynamically adapted to participants’ responses, the conversational structure, constraints, and thematic coverage remained identical across all participants and conditions. The chatbot did not provide feedback, advice, or interpretation and functional solely as a narrative elicitation interface.

### Measures

#### Psychological empowerment (Narrative-based)

Chatbot transcripts were segmented into semantically coherent narrative units. Each unit was deductively coded against Spreitzer’s [1] four empowerment dimensions and further analysed for valence (positive/neutral/negative), reflective depth (three-level scale: descriptive, interpretive, higher-order integrative [29,33], and attribution type (self, generalized, contextual) [34]. Three trained coders independently coded all transcripts, achieving high inter-rater reliability (Krippendorff’s α > 0.80).

#### Mental well-being

The LifeWork Well-being Scale, previously validated in Indonesian organizational settings [31], was used in this study. As part of measurement checks in the present sample, we conducted confirmatory analyses, which indicated adequate psychometric properties (factor loadings > 0.70, CR > 0.70, AVE > 0.50). Model fit indices were within acceptable ranges (CFI = 0.92; RMSEA = 0.079; SRMR = 0.074).

#### Leadership priming stimuli

Two 3-min video stimuli were developed to operationalize authentic leadership, based on [4]. The high-authenticity video depicted a leader demonstrating relational transparency, balanced processing, and moral integrity. The low-authenticity video portrayed a leader showing inauthentic cues such as vague communication and emotional incongruence. A neutral control video depicted generic workplace scenes without leadership cues. Expert validation confirmed strong content validity (Aiken’s V = 0.856 and 0.789). Both videos were recorded using the same actor, location, and script structure to control for extraneous variance.

### Data Analysis

#### Quantitative

Changes in well-being were assessed using gain scores, calculated as the difference between post-intervention and pre-intervention measures. A composite well-being gain score was also computed as a summary index to represent overall directional change across multiple dimensions, rather than as a reflection of a single latent construct.

This approach was selected as a pragmatic and interpretable method for estimating change across conditions in a pilot experimental design with a limited sample size. While gain scores do not account for the covariance structure between repeated measures or allow for analysis of interaction effects between time and group, they offer a direct summary of change suitable for exploratory comparisons. Differences across experimental groups were examined using analysis of variance (ANOVA), with Welch’s correction applied when the assumption of homogeneity of variance was violated. Assumptions of normality and homogeneity were evaluated prior to analysis, and post hoc comparisons were conducted as appropriate.

#### Qualitative

Narrative data from chatbot transcripts were analysed using thematic analysis [35] within a structural coding framework aligned with Spreitzer’s four psychological empowerment dimensions: meaning, competence, self-determination, and impact. In addition to this thematic categorization, two additional unit-level coding schemes applied to each narrative unit. First, narrative attribution and positioning were coded following Bamberg’s framework, which classifies narrative units as self-attributed, contextual, or generalized [34]. Second, reflective depth was coded from [29, 33], distinguishing between [1] descriptive reflection (Level 1) [2], interpretive/analytical reflection (Level 2), and [3] critical or higher-order integrative reflection (Level 3). When multiple levels of reflection were present within a single narrative unit, the highest level was recorded. All transcripts were independently coded by two trained coders. Inter-rater reliability exceeded recommended thresholds, with Krippendorff’s alpha values above 0.80 across all coding dimensions, indicating satisfactory coding consistency.

#### Integration

Findings from quantitative and qualitative analyses were integrated using a triangulation approach, linking changes in well-being outcomes with patterns of narrative empowerment expression. This integration was used to examine convergence and divergence between outcome-level changes and process-level narrative patterns, rather than to claim causal mediation. For transparency and replicability, detailed system-level instructions, fixed interaction constraints, conversational flow structure, and example prompt mapped to psychological empowerment dimensions are provided in Appendix A.

Psychological empowerment was assessed narratively via chatbot transcripts, while mental well-being was measured using the LifeWork Well-being Scale. Authentic leadership priming was operationalized through video-based stimuli. A summary of the study’s key measures, operational definitions, and validation indices is provided in Table 2.

**Table 2:** Summary of constructs, measures, and validation

Construct / Measure	Source / Basis	Operationalization	Reliability / Validity
Psychological Empowerment (Narrative-based)	[1, 29, 33, 34]	Chatbot transcripts segmented into narrative units; coded for 4 dimensions + valence, reflective depth, attribution	Krippendorff’s α > 0.80
Mental Well-being	Adapted LifeWork Well-being Scale [31]	4 subscales: purpose in life, workplace well-being, psychosomatic complaints, general well-being	CFI = 0.92; RMSEA = 0.079; SRMR = 0.074
Leadership Priming Stimuli	[4]	3-min high- vs. low-authenticity leadership videos; neutral workplace control video	Aiken’s V = 0.856 (high), 0.789 (low)
Reflective Chatbot Prompt Structure	Spreitzer’s [1] narrative elicitation frameworks	Standardized chatbot script of 4 sequential prompt blocks aligned to empowerment dimensions (meaning, competence, self-determination, impact); each block includes 2–3 indirect, culturally adapted reflective prompts	Script consistency ensured via fixed sequence; pilot testing (*n* = 12) confirmed clarity and cultural fit

## Results

### Quantitative findings: mental well-being gains

Descriptive statistics for pre-test, post-test, and gain scores across four dimensions of well-being purpose in life, workplace well-being, psychosomatic complaints, and general well-being are presented in Table 3. Participants in the High Authentic Leadership group (EG-1) demonstrated the most substantial improvements across most dimensions. Notably, workplace well-being increased by 4.50 points and purpose in life by 2.45 points, indicating that authentic leadership priming may enhance both affective and motivational aspects of well-being.

**Table 3:** Descriptive statistics of pre-test, post-test, and gain scores

Group	*N*	Pre-Test Mean (SD)	Post-Test Mean (SD)	Gain Score Mean (SD)
EG-1 (High AL)				
1. General well-being	18	18.56 (3.76)	22.44 (3.29)	3.88
2. Purpose in Life	18	11.61 (2.06)	14.06 (1.30)	2.45
3. Workplace Well-being	18	22.33 (4.17)	26.83 (2.53)	4.50
4. Psychosomatic complaints	18	6.89 (2.45)	7.67 (3.93)	0.78
EG-2 (Low AL)				
1. General well-being	17	20.06 (2.90)	18.00 (3.04)	-2.06
2. Purpose in Life	17	12.71 (1.79)	11.71 (2.23)	-1.00
3. Workplace Well-being	17	23.65 (3.46)	20.35 (4.33)	-3.30
4. Psychosomatic complaints	17	8.47 (3.32)	5.71 (2.71)	-2.76
CC (Neutral)				
1. General well-being	18	18.06 (3.64)	19.17 (3.33)	1.11
2. Purpose in Life	18	11.89 (2.54)	12.50 (2.57)	0.61
3. Workplace Well-being	18	22.78 (4.17)	23.39 (4.24)	0.61
4. Psychosomatic complaints	18	7.72 (1.84)	6.17 (2.50)	-1.55

By contrast, participants exposed to Low Authentic Leadership (EG-2) experienced declines in nearly all dimensions. General well-being decreased by 2.06 points and workplace well-being by 3.30 points, suggesting potential psychological costs of inauthentic leadership cues. Meanwhile, the Control group (CC), which interacted only with the chatbot, exhibited modest improvements in purpose in life and general well-being and a reduction in psychosomatic complaints.

Overall, the descriptive results suggest a graded pattern, with the High Authentic Leadership condition showing the most positive changes, the Low Authentic Leadership condition showing the least favourable changes, and the Control group falling in between. Notably, the pattern for psychosomatic complaints did not fully align with the graded trend observed in other well-being dimensions, suggesting a more complex or differential response across outcome types.

### ANOVA results: group differences in gain score

Descriptive comparisons across experimental conditions revealed that participants in the High Authentic Leadership group demonstrated consistent increases from pre-test to post-test in all well-being dimensions. In contrast, the Low Authentic Leadership group exhibited declines in most outcomes, while the Control group showed modest improvements in selected dimensions.

Assumption testing indicated that normality was satisfied (Shapiro–Wilk, *p* = .073), while homogeneity of variance was violated (Levene’s test, *p* = .004). Accordingly, Welch’s ANOVA, which is robust to unequal variances, was employed to examine differences in gain scores (post–pre differences) across experimental conditions. The analysis revealed significant group differences across all outcomes (see Table 4). However, the magnitude of these effects varied by dimension, with the strongest and most consistent effects observed for composite well-being (total gain), general well-being, and workplace well-being.

**Table 4:** One-way ANOVA results for gain scores across experimental conditions

Outcome	df (Group, Error)	F	*p*	η²
Composite well-being (total gain)	(2, 50)	56.40	< 0.001	0.69
General well-being (WHO)	(2, 50)	14.80	< 0.001	0.37
Workplace well-being	(2, 50)	22.30	< 0.001	0.47
Purpose in life	(2, 50)	10.40	< 0.001	0.29
Psychosomatic complaints	(2, 50)	5.00	0.010	0.17

For composite well-being, which is presented as a summary indicator, the omnibus test was significant, F (2, 50) = 56.4, *p* < .001. Holm-adjusted post hoc comparisons (Table 5) indicated that the high authentic leadership group showed significantly greater gains than both the low group (MD = 20.73, *p* < .001) and the control group (MD = 10.83, *p* < .001). Additionally, the control group outperformed the low group (MD = 9.90, *p* < .001).

**Table 5:** Pairwise comparisons between groups (Holm-adjusted p-values)

Outcome	Comparison	Mean difference	SE	t	*p* (Holm)
Composite Well-being (Total gain score)	High - Low	20.73	1.95	10.01	< 0.001
	High - Control	10.83	1.92	5.63	< 0.001
	Low - Control	-9.90	1.95	-5.07	< 0.001
General Well-being	High - Low	5.95	1.09	5.44	< 0.001
	High - Control	2.78	1.08	2.58	0.013
	Low - Control	-3.17	1.09	-2.90	0.011
Purpose in Life	High - Low	3.44	0.76	4.55	< 0.001
	High - Control	1.83	0.75	2.46	0.035
	Low - Control	-1.61	0.76	-2.13	0.038
Workplace Well-being	High - Low	7.79	1.17	6.68	< 0.001
	High - Control	3.89	1.15	3.38	0.003
	Low - Control	-3.91	1.17	-3.35	0.003
Psychosomatic Complaints	High - Low	3.54	1.14	3.10	0.010
	High - Control	2.33	1.13	2.07	0.087
	Low - Control	-1.21	1.14	-1.06	0.295

All p-values are Holm-adjusted. Positive mean differences indicate higher gains in the first group

A similar pattern was observed for general well-being, F (2, 50) = 14.8, *p* < .001. The high group demonstrated significantly larger gains than both the low group (MD = 5.95, *p* < .001) and the control group (MD = 2.78, *p* = .013). The control group also surpassed the low group (MD = 3.17, *p* = .011).

For the purpose in life, the omnibus effect was significant, F (2, 50) = 10.4, *p* < .001. Holm-adjusted post hoc tests revealed that the high group achieved greater gains than both the low group (MD = 3.44, *p* < .001) and the control group (MD = 1.83, *p* = .035). Furthermore, the control group demonstrated higher gains than the Low group (MD = 1.61, *p* = .038).

For workplace well-being, a significant group effect was observed, F (2, 50) = 22.3, *p* < .001. The high group outperformed both the low group (MD = 7.79, *p* < .001) and the control group (MD = 3.89, *p* = .003). The control group also exceeded the low group (MD = 3.91, *p* = .003).

Finally, for psychosomatic complaints, the omnibus test was significant, F (2, 50) = 5.00, *p* = .010. Holm-adjusted comparisons indicated that the high group differed significantly from the low group (MD = 3.54, *p* = .010), while differences involving the control group were not statistically significant (ps > 0.08).

Taken together, these results suggest a graded pattern across outcomes, although the magnitude and consistency of group differences varied by dimension. The High Authentic Leadership condition produced the largest and most consistent improvements, particularly for composite well-being, general well-being, and workplace well-being, whereas effects for purpose in life and psychosomatic complaints were more selective.

### Qualitative insights from chatbot reflections

Thematic analysis of the chatbot transcripts revealed distinct patterns of psychological empowerment expression across the experimental groups. Participants’ reflections were organized according to the four core dimensions of empowerment: meaning, self-determination, competence, and impact [1]. While all groups interacted with the same chatbot prompts, their narratives’ emotional tone, interpretive richness, and ownership varied notably depending on the leadership priming condition.

In their responses, participants in the High Authentic Leadership group (EG-1) consistently demonstrated a stronger sense of personal agency and meaning. Their narratives frequently conveyed a sense of purpose at work, linking daily tasks to personal values or long-term goals. For instance, one participant described how their demanding job, while stressful, felt meaningful because it contributed to both income and self-actualization. Such reflections not only affirmed task value but also signalled deeper motivational engagement. In contrast, participants in the Low Authentic Leadership condition (EG-2) often framed their motivation in terms of external pressures or survival goals. Their responses were more transactional in tone, reflecting disengagement or obligation rather than intrinsic purpose. The Control group (CC), which did not receive any leadership stimulus, expressed a narrower sense of meaning typically associated with task completion or service delivery indicating a more procedural or duty-bound orientation.

Similar patterns emerged in the self-determination dimension. Participants in EG-1 described autonomous problem-solving strategies and expressed confidence in navigating work challenges. They reported feeling supported by leadership, which enabled them to take the initiative and set priorities effectively. In contrast, participants in EG-2 shared experiences of uncertainty and diminished agency, often attributing this to unsupportive or volatile leadership behaviour. Their responses reflected a loss of confidence and a reliance on external validation. Meanwhile, participants in the control group acknowledged their dependence on hierarchical structures to complete tasks, framing self-determination less as a personal resource and more as a product of organizational protocols.

The competence domain further distinguished the groups. EG-1 participants articulated a clear sense of capability, often referencing successful task execution or mentoring roles. These reflections conveyed pride and readiness to contribute more actively. By comparison, EG-2 participants expressed frustration when their input was dismissed or undervalued, with some reporting that their suggestions were ignored without explanation. It led to a perceived gap between their actual ability and how they were treated. The control group offered more conditional expressions of competence, typically indicating confidence only in areas of prior expertise.

Finally, in the impact domain, EG-1 participants described collaborative problem-solving and how their contributions made a meaningful difference to their teams. They emphasized mutual support and shared goals. EG-2 reflections, however, tended to centre on performance metrics or customer satisfaction without linking these outcomes to a personal initiative or influence, suggesting a more passive role. Participants in the control group described the impact as dependent on external circumstances, such as the leader’s mood or situational dynamics, highlighting the absence of perceived influence over outcomes.

Together, these qualitative insights suggest that authentic leadership priming is associated with differences in the depth, tone, and ownership of empowerment narratives. Authentic leadership is associated with richer and more self-referential storytelling by shaping the cognitive-emotional frame through which employees interpret their work, especially when combined with reflective tools like chatbots.

To illustrate these thematic differences, Table 6 presents representative narrative excerpts from participants across the three experimental conditions, categorized by empowerment dimension. These samples demonstrate how leadership priming shaped the content of empowerment reflections and their tone, structure, and level of personal ownership.

**Table 6:** Sample reflections by group and empowerment dimension

Dimension	EG-1 (High Authentic Leadership)	EG-2 (Low Authentic Leadership)	CC (Neutral)
Meaning	“Although the high demand can be stressful, this job is meaningful to me because it provides income and opportunities for self-actualization” (E19).	“I find motivation in this challenging job because of my goal to achieve financial independence as part of the sandwich generation. I just try to do my best” (E12)	“I feel meaningful when the services provided to employees are completed thoroughly and on time” (C5).
Self-determination	“I feel responsible for my work and will try my best. I set priorities, make a to-do list, and focus on completing it to the maximum” (E18)	“I used to feel capable, but because my boss often gets angry and complains, now I somewhat doubt my abilities” (E4)	“I need leadership guidance to complete the task, because our highest leader is the Head of Echelon 2 Centre, assisted by the Head of the Work Team” (C3).
Competence	“I feel more confident in handling problems in the ordering department and am ready to immediately mentor new employees” (E26).	“I have tried to provide solutions based on my experience dealing with customer complaints, but my opinions were rejected without further discussion or direction.” (E8)	“My level of confidence in collaborating depends on the area. If it is within my expertise, I would rate my confidence an 8 out of 10” (C2).
Impact	“I feel capable of solving challenges by collaborating with my team and supporting each other to find solutions” (E6)	“Customer happiness has a positive impact on my mood and motivation. Meeting targets and improving quality gives me satisfaction, even though external perceptions are beyond my control” (E24).	“The quality of the discussion depends on who is leading it. If the boss is in a good mood, the discussion feels warm and interesting: D” (C1).

### Narrative valence and reflective depth

To further explore the quality of reflective engagement, each narrative unit was coded for valence (positive, neutral, or negative) and reflective depth, following a three-level scale: Level 1 (descriptive), Level 2 (interpretive), and Level 3 (higher-order integrative reflection) (see Table 7). Participants in the High Authentic Leadership (EG-1) group most frequently generated positively valenced narratives and demonstrated deeper cognitive processing. Their reflections were often future-oriented, grounded in self-confidence, and expressed with emotional richness hallmarks of Level 2 and 3 responses. In contrast, participants in the Low Authentic Leadership (EG-2) condition produced a higher proportion of negatively valenced reflections, often conveying frustration, disengagement, or a sense of diminished agency. Their narratives tended to remain at the surface level or only moderately interpretive. Meanwhile, the Control group (CC) mainly produced neutral or emotionally flat responses, with reflections concentrated at Level 1 suggesting basic task-focused description with minimal introspection.

**Table 7:** Distribution of narrative valence and reflective depth across empowerment dimensions

Psychological Empowerment Dimensions	Group	Positive (%)	Neutral (%)	Negative (%)	Level 1 (%)	Level 2 (%)	Level 3 (%)
Meaning	EG-1	100,0	0,0%	0,0%	1,5	55,2	43,3
	EG-2	89,8	3,4	6,8	5,1	78,0	16,9
	CC	96,1	1,3	1,3	3,9	63,2	31,6
Competence	EG-1	97,7	2,3	0,0%	3,4	63,2	33,3
	EG-2	90,9	3,9	5,2	6,5	58,4	35,1
	CC	95,2	1,2	2,4	7,2	69,9	21,7
Self-determination	EG-1	96,3	2,4	1,2	6,1	67,1	26,8
	EG-2	79,1	4,7	16,3	5,8	80,2	14,0
	CC	88,5	9,4	1,0	12,5	59,4	27,1
Impact	EG-1	94,4	4,5	1,1	4,5	67,4	28,1
	EG-2	80,3	13,2	6,6	7,9	78,9	13,2
	CC	93,7	5,1	0,0%	6,3	84,8	7,6

Inter-rater reliability for both coding dimensions was high, with Krippendorff’s alpha values of 0.87 for valence and 0.84 for depth, indicating consistency in rating judgments across coders. As shown in Table 7, interpretive reflections (Level 2) dominated across all groups and empowerment dimensions, indicating that participants generally engaged in moderate cognitive processing during their reflections. However, the EG-1 group consistently demonstrated a higher proportion of higher-order integrative reflection (Level 3) compared to the other groups, particularly in the domains of Meaning (43.3%) and Competence (33.3%). By contrast, the EG-2 group showed high engagement at Level 2 across dimensions such as Self-Determination (80.2%) and Impact (78.9%) but rarely progressed into deeper, higher-order integrative reflection (Level 3). The Control group (CC) also reflected this trend, with reflections largely anchored at Level 2 and limited signs of deeper introspection, especially in Impact (only 7.6% Level 3). These patterns suggest that authentic leadership priming supports not only the emotional quality of reflection but also a tendency toward more elaborated and integrative reflective engagement, even when Level 2 responses remain the most prevalent across conditions. It should be noted, however, that Level 2 responses remained dominant across all conditions, and Level 3 constituted a minority of responses even in the high authentic leadership group.

### Narrative Attribution Pattern

Beyond emotional tone and reflective depth, empowerment narratives were analysed for their attribution structure to elucidate how participants positioned themselves within their reflections. Using Bamberg’s [34] positioning framework, narrative units were categorized as self-attributed, contextual, or generalized. Self-attributed reflections were rooted in personal experience and self-evaluation, reflecting internal ownership of psychological states. Contextual narratives referenced specific work settings or organizational processes without directly implicating the self. Generalized narratives expressed impersonal or collective sentiments, remaining detached from individual experience.

Table 8 demonstrated that self-attributed narratives predominated across all groups and empowerment dimensions, indicating a strong tendency for participants to anchor in personal experiences. Distinct patterns were observed between groups. The High Authentic Leadership (EG-1) group exhibited a high proportion of self-attributed reflections in Meaning (89.6%) and Competence (90.8%). However, a significant portion of their Impact reflections (23.6%) were contextually framed, suggesting increased awareness of situational factors influencing perceived impact.

**Table 8:** Narrative attribution types by experimental group and empowerment domain

Psychological Empowerment Dimension	Group	Self-attributed (%)	Contextual (%)	Generalized (%)
Meaning	EG-1	89,6	6,0	4,5
	EG-2	86,4	8,5	5,1
	CC	96,1	1,3	1,3
Competence	EG-1	90,8	8,0	1,1
	EG-2	85,7	11,7	2,6
	CC	94,0	2,4	2,4
Self-determination	EG-1	86,6	11,0	2,4
	EG-2	88,4	10,5	1,2
	CC	85,4	10,4	3,1
Impact	EG-1	70,8	23,6	5,6
	EG-2	65,8	32,9	1,3
	CC	86,1	11,4	1,3

The Low Authentic Leadership (EG-2) group also demonstrated high self-attribution in Self-Determination (88.4%) and Meaning (86.4%). However, they showed a greater tendency toward contextual framing, particularly in the Impact domain (32.9%), compared to EG-1 and the control group. This pattern indicates that, although participants engaged with the reflective prompts, their narratives more frequently positioned agency externally rather than fully internalizing it.

The Control group (CC) exhibited consistently high self-attribution in Meaning (96.1%) and Competence (94.0%), with relatively few contextualized reflections overall. An exception was observed in the Impact domain (11.4%), where some external references were used. Collectively, these patterns suggest that authentic leadership priming facilitated more balanced and nuanced reflective engagement. Participants in the EG-1 group integrated personal ownership with contextual awareness, particularly when reflecting on perceived impact. In contrast, the EG-2 group, while engaging in self-attribution, exhibited greater psychological distancing in domains associated with external factors, indicating a less internalized sense of empowerment. These findings support the interpretation that authentic leadership priming influences not only the emotional tone and cognitive depth of reflective narratives but also how participants position themselves as agents within their work experiences.

### Integrated Insights

Collectively, the findings demonstrate that leadership priming is associated with differences in both quantitative well-being outcomes and the qualitative structure of narrative empowerment. The High Authentic Leadership (EG-1) group exhibited the most favourable outcomes, including greater improvements in well-being and narratives that were more emotionally engaged, future-oriented, and grounded in personal meaning. Reflections from this group were primarily characterized by interpretive (Level 2) processing, with a higher, though still minority, proportion of higher-order integrative reflection (Level 3) narratives, particularly within the domains of meaning and competence. Additionally, these narratives more frequently reflected self-attributed ownership while still acknowledging contextual factors, especially in discussions of impact.

By contrast, the Low Authentic Leadership (EG-2) group demonstrated a distinct pattern. While their narratives often achieved interpretive (Level 2) engagement, particularly in the domains of Self-Determination and Impact, they seldom advanced to higher-order integrative reflection (Level 3). These accounts also exhibited a stronger tendency toward contextual framing, especially in the impact domain, indicating a less internalized and more extremely anchored sense of urgency.

The Control group (CC) showed modest improvements in both well-being and narrative engagement. Their reflections were generally neutral or affectively restrained and were primarily situated in the interpretive (Level 2) level, with minimal evidence of deeper higher-order integrative reflective processing, particularly in the impact domain.

Taken together, these patterns suggest that exposure to authenticity-related leadership cues may serve as a psychological context-setting factor influencing the approach and articulation of AI-guided reflection. The observed group differences are primarily descriptive and vary in magnitude across outcome domains. Rather than indicating categorical changes in reflective capacity or narrative form, the findings highlight subtle yet systematic variations in how participants frame meaning, agency, and psychological resources during reflection.

## Discussion

### Authentic leadership priming and reflective chatbots: influencing empowerment narratives and well-being in digitally mediated workplace

This study investigated the interaction between authentic leadership priming and AI-guided reflective chatbots in shaping empowerment narratives and supporting well-being within digitally mediated workplaces. Drawing on psychological empowerment theory [1], social cognitive leadership models [19], and recent research on AI-mediated reflection, including self-disclosure chatbots that foster trust and conversational depth [24] and structured self-reflection chatbots [25], the findings indicate that leadership cues and digital reflection tools together create a context that supports and structures the exploration and articulation of meaning, agency, and self-insight. However, the results should be interpreted with caution, as the observed narrative differences across conditions are descriptive and modest in magnitude. However, the results should be interpreted with caution, as the observed narrative differences across conditions are descriptive and modest in magnitude. The qualitative findings are intended to provide descriptive and interpretive insights into participants’ reflective experiences, rather than to develop new theoretical constructs. In contrast, the quantitative well-being outcomes offer more robust empirical evidence of intervention effects.

### Leadership cues and AI-guided reflection

Regarding H1, participants in the high-authentic leadership condition (EG-1) tended to produce narratives that were more emotionally engaged, future-oriented, and self-referential compared to those in the low-authentic leadership (EG-2) and control (CC) conditions. However, Level 3 (higher-order integrative reflection) reflections were present across all groups, and their distribution suggests only modest differences rather than categorical shifts in reflective depth. These findings indicate variations in reflective framing rather than the emergence of distinctly narrative modes.

Previous research on charismatic and relational leadership demonstrates that leaders’ emotional expression and sense giving influence followers’ affective states and interpretive frames [36, 37]. Accordingly, the richer emotional tone observed in some EG-1 narratives and at the aggregate level aligns with broader social information processing processes [38], rather than indicating a deterministic causal mechanism. In contrast, narratives in EG-2 and CC were more frequently descriptive or context-focused, which likely reflects differences in participants’ approaches to the reflective task under varying priming conditions rather than differences in inherent reflective capacity.

### Narrative ownership and psychological empowerment

Attribution analysis revealed that self-attributed narratives predominated across all groups, with some variation in the balance between self and contextual attribution. Participants in EG-1 typically combined strong self-referential framing in meaning and competence with some contextual framing in impact, while EG-2 narratives exhibited a greater amount of contextualized framing. These differences are descriptive and modest in magnitude and should be interpreted as indicative patterns rather than evidence of qualitatively distinct forms of narrative agency.

From a theoretical perspective, these patterns align with psychological empowerment theory and self-determination perspectives, which emphasize meaning, competence, and autonomy as key factors supporting intrinsic motivation and self-reflection [39]. In this study, leadership priming influenced how participants framed and articulated their experiences during reflection, rather than indicating any fundamental alteration in the underlying structure of their narratives.

### Synergy between reflection and well-being

The integration of quantitative well-being outcomes with narrative patterns offers converging evidence supporting H2. Participants in EG-1 demonstrated the largest improvements across well-being indicators, while EG-2 experienced declines, and the control group exhibited more modest changes. These results indicate an association between reflective engagement under supportive leadership cues and psychological resources, rather than a direct causal relationship between specific narrative forms and changes in well-being.

This pattern aligns with previous research indicating that authentic and supportive leadership correlates with higher intrinsic motivation, greater satisfaction of basic psychological needs, and reduced exhaustion [40, 41]. In this study, participants exposed to supportive leadership cues appeared to approach the reflective task with a more constructive and resource-oriented mindset, as reflected in both their well-being scores and narrative tone. In contrast, the declines observed in EG-2 are consistent with resource loss perspectives, such as the Conservation of Resources Theory [42], which posits that unsupportive cues can undermine psychological resources and engagement. The control group’s modest gains suggest that chatbot-guided reflection alone may foster basic engagement, but that the leadership context appears to meaningfully shape the experience of such reflection. The divergence observed in psychosomatic complaints suggests that physiological or stress-related indicators may respond differently to short-term interventions compared to affective or meaning-related dimensions of well-being. This indicates that not all well-being dimensions follow a uniform pattern of change, particularly in brief, reflection-based interventions.

The relatively large effect sizes observed in this study may reflect the controlled experimental context and the salience of the leadership manipulation. However, these effects should be interpreted with caution given the modest sample size, particularly in a multi-group design.

### The moderating role of leadership cues in chatbot reflection

With respect to H3, chatbot-guided reflection elicited empowerment-related narratives across all conditions, with descriptive differences in depth and framing influenced by leadership context. In EG-1, narratives more often included future-oriented and emotionally elaborated elements, consistent with CASA paradigm, which posits that socially present and empathic chatbots can foster greater engagement and disclosure [43–45]. In EG-2 and CC, reflections were more often descriptive, indicating that AI-mediated reflection is sensitive to leadership context rather than functioning as a context-free intervention.

Previous studies demonstrate that positive leader-follower relationships, characterized by active support, clear goal communication, and encouragement to challenge the status quo, are associated with higher perceptions of psychological safety. This, in turn, relates to speaking up, experimentation, and learning-oriented behaviours [46]. More broadly, interpersonal processes such as active listening and relational depth support emotional engagement and meaning-making in human interaction [39, 47]. However, as psychological safety and related interpersonal processes were not directly assessed in this study, these constructs should be considered as theoretical interpretive perspectives and potential avenues for future research, rather than empirically tested mechanisms.

### Theoretical implications

This study contributes to the literature in three main ways. First, it extends psychological empowerment research by integrating narrative characteristics (valence, depth, and attribution) with quantitative well-being outcomes, thereby offering a more process-oriented and interpretive perspective on empowerment in digital contexts. Rather than treating empowerment solely as an attitudinal state, this approach highlights how empowerment is articulated and negotiated through reflective narratives.

Second, this study advances understanding of leadership–technology synergy by showing how authentic leadership cues shape the way employees engage with AI-guided reflective tools, rather than treating technology as a context-free intervention. Methodologically, this is achieved by combining experimental leadership priming with AI-guided reflection, offering a scalable and data-driven approach for examining empowerment processes in digitally mediated workplaces. The findings suggest that leadership cues influence not only outcomes, but also how individuals approach and interpret digitally mediated reflection.

Third, this study contributes to cross-cultural organizational psychology by demonstrating how these processes unfold in a collectivist, high power-distance context such as Indonesia, where empowerment may be experienced but not always openly articulated [16, 48]. This highlights the importance of considering cultural scripts and normative expectations when theorizing about digital reflection and leadership effects.

Taken together, the findings suggest a dual-pathway process in which leadership priming shapes the interpretive framing of experience, while AI-guided reflection structures the articulation of that experience. These pathways jointly contribute to the development of empowerment narratives and associated well-being outcomes.

### Practical implications

The findings indicate that AI-guided reflective chatbots may serve as cost-effective, scalable tools to facilitate employee reflection, especially when combined with leadership communication that prioritizes transparency, respect, and a future-oriented approach. The results further emphasize that technology by itself is inadequate and highlight the necessity of integrating digital tools with effective leadership practices.

For instance, chatbot scripts can include empathetic language, value-driven prompts, and future-oriented questions that reflect constructive leadership communication. Within leadership development and digital human resource interventions, these tools may facilitate structured reflection, onboarding processes, or regular well-being assessments, particularly in organizations with limited access to coaching or psychological services.

More broadly, these findings suggest that organizations should consider AI-based reflective tools as integral elements of a comprehensive leadership and people development system, rather than as standalone technological solutions. In this context, digital reflection tools can influence how employees interpret and understand their work experiences, while remaining closely aligned with organizational leadership practices.

### Limitations and boundary conditions

Several limitations warrant consideration. First, the modest sample size limits statistical power, particularly for detecting interaction effects in a multi-group experimental design and restricts the generalizability of the findings. Second, the short-term nature of the intervention allowed for the assessment of only immediate reflective responses, rather than long term change. Third, self-generated narrative data may be influenced by demand characteristics or social desirability bias, especially within a chatbot-mediated context.

Fourth, the study examined a specific occupational and cultural group in Indonesia. While this context is theoretically significant, it represents a key boundary condition. Consequently, the findings should be interpreted with caution and not presumed to generalize to low power-distance or more individualistic organizational cultures without additional empirical research.

Fifth, the study did not include participant-level manipulation checks to confirm whether the leadership stimuli were perceived as intended. Although expert validation supported the content validity of the videos, future research should incorporate manipulation checks to strengthen internal validity.

Finally, the composite well-being score aggregates conceptually distinct dimensions and does not represent a validated higher-order construct. In addition, the use of gain scores simplifies change estimation but does not capture within-subject covariance or interaction effects. Therefore, these measures should be interpreted as summary indicators and exploratory estimates rather than precise representations of dynamic change processes.

### Future research directions

Future research should utilize longitudinal designs to assess the long-term stability of both narrative and well-being effects, replicate the methodology in varied cultural contexts, and incorporate larger, more heterogeneous samples. Additionally, subsequent studies should directly measure constructs such as psychological safety, trust, and speak-up behaviour to explicitly test their potential mediating or moderating roles, rather than relying on interpretive assumptions.

Furthermore, computational text analysis and natural language processing (NLP) methods could complement human coding to expand the scalability of narrative analysis and enable real-time organizational feedback systems in applied contexts.

## Conclusion

This study investigated the relationships among authentic leadership priming, AI-guided reflective chatbots, and employees’ empowerment-related narratives and well-being within a digitally mediated work environment. The results indicate that integrating leadership cues with digital reflection tools can foster conditions conducive to employees’ reflective sensemaking about meaning, agency, and psychological resources. Notably, differences in narrative patterns across experimental conditions were mainly descriptive and modest in size, while quantitative well-being outcomes offered stronger evidence of intervention effects.

From an applied perspective, the findings suggest that incorporating leadership-consistent signals, including empathetic language, value-oriented prompts, and future-focused framing, into digital reflection tools may provide a scalable method for supporting employee reflection and well-being, especially in settings with limited access to coaching or psychological services.

Given that the study was conducted in a collectivist, high power-distance context, the findings should be interpreted within these boundary conditions. Future research is needed to evaluate the cultural generalizability of these results, investigate longer-term effects in real-world workplace environments, and further elucidate the psychological mechanisms connecting leadership cues, digital reflection, and employee well-being.

## Supplementary Information

Supplementary Material 1.

## Data Availability

The datasets generated and analysed during the current study are not publicly available due to confidentiality restrictions but are available from the corresponding author upon reasonable request.
