# Supplementary material for: Activating empowerment through AI-guided reflection: the role of authentic leadership cues in a randomized controlled experiment
Source: BMC Psychol. 2026 Apr 14;14:771. doi: 10.1186/s40359-026-04537-y (PMC13188245; doi:10.1186/s40359-026-04537-y)
Supplement: Supplementary file 1 — Supplementary Material 1. [file 40359_2026_4537_MOESM1_ESM.docx]

**Appendix A**

**Chatbot-Based Reflective Protocol**

**A1. Experimental context and procedure**

Following the leadership priming procedure, participants engaged in a reflective conversation interaction with an AI chatbot (“Sarah”) via WhatsApp Web. This interaction was structured as a narrative elicitation task, guiding participants from reflecting on the observed video scenario to articulating their own work experiences and perceived psychological empowerment at work. The chatbot did not provide feedback or intervention but served solely as a medium to facilitate reflective self-expression in a psychologically neutral, non-evaluative manner.

**A2. System-level instruction**

Bahasa Indonesia (as used in the study):

*Anda adalah Sarah, seorang rekan kerja yang bersahabat. Teman anda baru saja mengikuti rapat bersama bosnya.*

*Pada setiap pesan yang kamu kirimkan, hanya boleh berisi satu pertanyaan dan berikan pertanyaan tersebut dengan gaya non formal secara natural dan tidak terlalu panjang. Galilah secara alamiah mengenai bagaimana suasana rapat di pekerjaan asli. Kemudian, alihkan percakapan secara natural untuk mengeksplorasi bagaimana perasaan mereka tentang pekerjaan mereka sendiri, terutama terkait dengan dimensi Psychological Empowerment mereka. Berikut adalah indikator2 dari psychological empowerment yang kamu perlu eksplorasi: - Competence (seberapa percaya diri mereka akan kemampuan mereka) - Meaning (seberapa bermakna/meaningful pekerjaan mereka bagi mereka) - Impact (seberapa besar dampak dan kontrol yang mereka miliki di tempat kerja dan pada pekerjaan mereka) - Self-determination (seberapa besar kemungkinan mereka untuk dapat menentukan cara kerja mereka dan menyelesaikan pekerjaan mereka).*

*Pastikan kamu menggali secara dalam, rinci, friendly, dan subtle.*

*Ingat, untuk setiap pesan yang kamu kirimkan, HANYA TANYA SATU PERTANYAAN.*

*Apapun itu pesannya, berilah jawaban pertamamu "Haloo, gimana pendapatmu tentang diskusi tadi?"*

English translation (for reference only; not shown to participants):

*You are Sarah, a friendly coworker. Your friend has just come out of a meeting with their boss.*

*In each message you send, only ask one question, and ask it in a natural, informal style that isn't too long. Explore naturally how the discussion atmosphere is at the participants' actual jobs. Then, naturally shift the conversation to explore how they feel about their own work, particularly regarding their Psychological Empowerment dimension. Here are some indicators of psychological empowerment you need to explore: - Competence (how confident they are in their abilities) - Meaning (how meaningful their work is to them) - Impact (how much impact and control they have in their workplace and over their work) - Self-determination (how likely they are to be able to determine how they work and complete their work). Make sure you probe deeply, thoroughly, kindly, and subtly.*

*Remember, for each message you send, ONLY ASK ONE QUESTION.*

*Whatever the message, give your first response: "Hello, what do you think about the discussion earlier?"*

**A3. Interaction constraints and conversational flow**

The chatbot was governed by fixed interaction constraints to ensure procedural consistency across all experimental sessions. It limited itself to asking one question per message, maintained a friendly, non-evaluative tone, and consistently initiated each interaction with a predetermined opening question. The conversation was structured to transition gradually from reflections on the video scenario to participants’ work experiences, addressing all four dimensions of psychological empowerment: meaning, competence, impact, and self-determination.

The interaction adhered to a semi-structured conversational flow comprising four phases: initial reflection on the video scenario and its discussion atmosphere, transition to the participant’s work context, in-depth exploration of the four psychological empowerment dimensions, and reflective consolidation and closure.

Although the wording of follow-up questions varied in response to participants’ answers, the thematic structure, sequencing logic, and interaction constraints were consistent across all sessions.

**A4. Standardized opening prompt**

In all sessions, the chatbot began each interaction with a standardized opening question, which functioned as a contextual link between the video stimulus and participants’ subsequent reflections:

“Halo, gimana rapatnya tadi?”

(“Hello, how was the meeting earlier?”)

**A5. Prompt framework mapped to empowerment dimensions**

While the interaction maintained a conversational tone rather than a strictly segmented structure, the prompts were designed to address four key dimensions of psychological empowerment. Representative prompt formulations included:

Table 1. Example Chatbot Prompts Mapped to Psychological Empowerment Dimensions

| **Dimension** | **Example prompts (English translation)** |
| --- | --- |
| Meaning | “What makes your work feel meaningful to you?” “Which part of your job feels most important to you personally?” |
| Competence | “How confident do you usually feel about handling your main tasks?” “Can you recall a time when you felt capable or not capable at work?” |
| Self-determination | “How much freedom do you feel you have in deciding how you do your work?” “Can you describe a situation where you could or could not choose how to work?” |
| Impact | “In what ways do you feel your work makes a difference?” “How much influence do you feel you have over what happens in your team?” |

The chatbot dynamically selected and sequenced prompts while adhering to the established thematic structure and constraints.

**A6. Language of interaction**

All chatbot interactions were conducted in Bahasa Indonesia, the participants' native language, to ensure linguistic authenticity, emotional nuance, and cultural relevance during reflective questioning. The prompts in this appendix are presented in the original Bahasa Indonesia. English translations are included solely for reference and were not provided to participants during data collection.

**A7. Replicability statement**

This appendix details the system-level instructions, fixed interaction constraints, conversational flow structure, and the prompt framework aligned with psychological empowerment dimensions. These specifications provide sufficient information to facilitate replication of the reflective chatbot procedure. Full verbatim chat logs and complete message sequences are available from the authors upon reasonable request.
